# High seroprevalence of selected vector-borne pathogens in dogs from Saipan, Northern Mariana Islands

**DOI:** 10.1186/s13071-025-06705-2

**Published:** 2025-02-24

**Authors:** Maureen A. Kelly, Kris Anderson, Meriam N. Saleh, Rafael A. N. Ramos, Robert J. Valeris-Chacin, Christine M. Budke, Guilherme G. Verocai

**Affiliations:** 1 Department of Veterinary Pathobiology, College of Veterinary Medicine and Biological Sciences, Texas A&M University, College Station, TX 77843 USA; 2 Equine Mobile Veterinary Services, Santa Fe, TX 77510 USA; 3 Laboratory of Parasitology, Federal University of the Agreste of Pernambuco, Garanhuns, PE 55292-278 Brazil; 4 Department of Veterinary Integrative Biosciences, College of Veterinary Medicine and Biomedical Sciences, Texas A&M University, College Station, TX 77843 USA

**Keywords:** Dog, Canine vector-borne disease, SNAP^®^ 4Dx^®^, Prevalence, Saipan, Epidemiology

## Abstract

**Background:**

Canine vector-borne diseases (CVBDs) are illnesses caused by pathogens transmitted by blood-feeding arthropods such as ticks and mosquitoes. Many CVBDs, including dirofilariosis, anaplasmosis, and ehrlichiosis, are globally distributed and may cause a variety of clinical signs in dogs. Several CVBD agents are zoonotic, making epidemiological surveillance a joint veterinary and public health effort. In this study, we determined the seropositivity of four pathogens from dogs on Saipan, Northern Mariana Islands, a US Commonwealth located in the western Pacific Ocean.

**Methods:**

Blood samples (*n* = 443) were collected from client-owned, owner surrendered, and shelter dogs that participated in an island-wide spay-and-neuter event in 2023. All samples were assessed using a commercial, point-of-care enzyme-linked immunosorbent assay (ELISA) test (SNAP^®^ 4Dx^®^ Plus, IDEXX Laboratory, Westbrook, Maine, USA) to detect the *Dirofilaria immitis* antigen and antibodies against *Ehrlichia* spp., *Anaplasma* spp., and *Borrelia burgdorferi* sensu lato. Risk factors were assessed for each pathogen through a univariate analysis, followed by a multivariable logistic regression.

**Results:**

Overall, 66.1% (*n* = 300/443) of the dogs tested positive for at least one pathogen, with the highest prevalence observed for *Ehrlichia* spp. (58.0%; *n* = 246/443), followed by *Anaplasma* spp. (43.1%; *n* = 184/443) and *D. immitis* (14.8%; *n* = 63/443). Among the dogs with a single pathogen detected (30.9%; *n* = 137/443), *Ehrlichia* spp. was most prevalent (64.9%; *n* = 89/137), followed by *Anaplasma* spp. (23.3%; *n* = 32/137) and *D. immitis* (11.6%; *n* = 16/137). For co-detection of two or more pathogens (36.7%; *n* = 163/443), *Ehrlichia* spp. + *Anaplasma* spp. presented the highest frequency (70.5%; *n* = 115/163), followed by *Ehrlichia* spp. + *D. immitis* (6.7%; *n* = 11/163), *Anaplasma* spp. + *D. immitis* (3.6%; *n* = 6/163), and *Ehrlichia* spp. + *Anaplasma* spp. + *D. immitis* (19.0%; *n* = 31/163). Age (*P* = < 0.001), residing district (*P* = 0.001), and ownership status (*P* = < 0.001) were significantly associated with *D. immitis* positive status in a univariable analysis. Age (*P* = < 0.001), residing district (*P* = 0.177), and ownership status (*P* = 0.014) were significant in a univariable analysis with *Ehrlichia* spp. as an outcome. Finally, *Anaplasma* spp. had a significant association with ownership status (*P* = < 0.001) as a risk factor in a univariable analysis.

**Conclusions:**

This study shows high seropositivity for CVBPs in a dog population living in a poorly studied area. The results of this study suggest that strategies for the prevention and control of these CVBDs should be reinforced on the Island of Saipan.

**Graphical Abstract:**

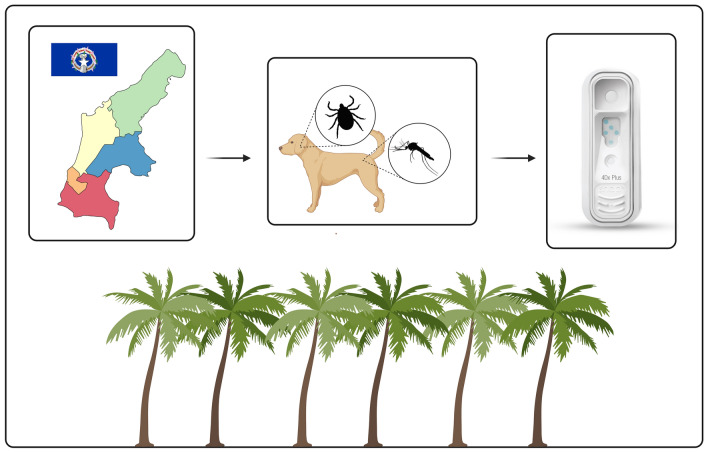

**Supplementary Information:**

The online version contains supplementary material available at 10.1186/s13071-025-06705-2.

## Background

Vector-borne pathogens (VBPs) include parasites, bacteria, and viruses that can cause a variety of clinical manifestations in animals and people [1–5]. These pathogens are transmitted by blood-feeding arthropods such as ticks, fleas, sand-flies, and mosquitoes [2, 3]. Globally, these vectors are directly related to the presence and abundance of VBPs, accounting for more than 17% of all infectious diseases [1, 2, 6–10]. The major VBPs known to infect dogs include *Dirofilaria* spp., *Anaplasma* spp., and *Ehrlichia* spp., which are commonly known as canine vector-borne pathogens (CVBPs). These pathogens have a wide range of vector species with varying distributions but are predominantly found in tropical and subtropical regions.

*D. immitis* is a zoonotic, mosquito-borne filarial nematode, and the causative agent of canine heartworm disease. This parasite is transmitted by approximately 60 different mosquito species, with those belonging to the genera *Aedes*, *Anopheles,*, and *Culex* having importance epidemiologically [11–16]. Domestic dogs are the definitive hosts, and despite many asymptomatic cases, the chronic progression of disease is often fatal owing to the presence of adult worms in the pulmonary arteries and right ventricle of the heart [17–20]. Current diagnostics guidelines from the American Heartworm Society (AHS) and other veterinary advisory boards recommend annual testing of dogs over 7 months of age with a combination of two tests, a serology-based test to detect heartworm antigen and a microfilariae detection test [19–22].

*Ehrlichia* spp. and *Anaplasma* spp. are tick-borne bacterial pathogens, with some species of ticks such as the brown dog tick, *Rhipicephalus sanguineus* sensu lato (s.l.), playing a more substantial role in transmission globally [23]. *Ehrlichia* species infecting dogs include *Ehrlichia canis*, *Ehrlichia chaffeensis*, and *Ehrlichia ewingii* [24, 25]. *E. canis* is transmitted by *R. sanguineus* s.l. and is found worldwide, whereas *E. ewingii* and *E. chaffeensis* are found in the southeast and south-central continental USA and are commonly vectored by *Amblyomma americanum* [23, 26]. *E. ewingii* and *E. chaffeensis* have been identified in dogs and humans on other continents, including Asia and Europe; however, these studies were based on serological testing and did not focus on vector transmission [8, 27]. Commonly known as canine monocytic ehrlichiosis (*E. canis*) and canine granulocytic ehrlichiosis (*E. ewingii*), these diseases are characterized by fever, diarrhea, and vomiting, with potential to be fatal [24, 26, 28]. *Ehrlichia* spp. have been documented on all continents, especially in tropical and subtropical areas that are favorable to the brown dog tick. Diagnostics include commercially available serology-based immunofluorescence antibody (IFA) and immunochromatography-based point-of-care (POC) tests, as well as polymerase chain reaction (PCR) tests as confirmatory tests [24, 25, 29].

In dogs, *Anaplasma phagocytophilum* and *Anaplasma platys* are both transmitted by multiple genera of ixodid ticks, including *Rhipicephalus*, *Ixodes*, *Dermacentor*, *Haemaphysalis*, and *Amblyomma* [23, 30–33]. Although both of these *Anaplasma* species are known to be zoonotic, *A. platys* is not frequently reported in humans [34, 35]. *A. phagocytophilum* is the causative agent of granulocytotropic anaplasmosis in dogs, in combination with other common clinical signs of VBPs such as fever, lethargy, and joint swelling [33]. However, *A. platys* is clinically featured by the presence of cyclic thrombocytopenia presenting with similar clinical signs of *A. phagocytophilum*; nonetheless, distinction between these two pathogens is crucial [26]. Diagnostic techniques are similar to those used for other tick-borne pathogens and include IFAs, POC tests, and PCR [24, 25].

There has been limited surveillance of CVBDs in Saipan or any of the Commonwealth Northern Mariana Islands (CNMI). Therefore, the aim of this study was to investigate the prevalence of CVBPs in dogs on the island of Saipan and to assess possible risk factors associated with exposure to these pathogens.

## Methods

### Study area

**Fig. 1:**
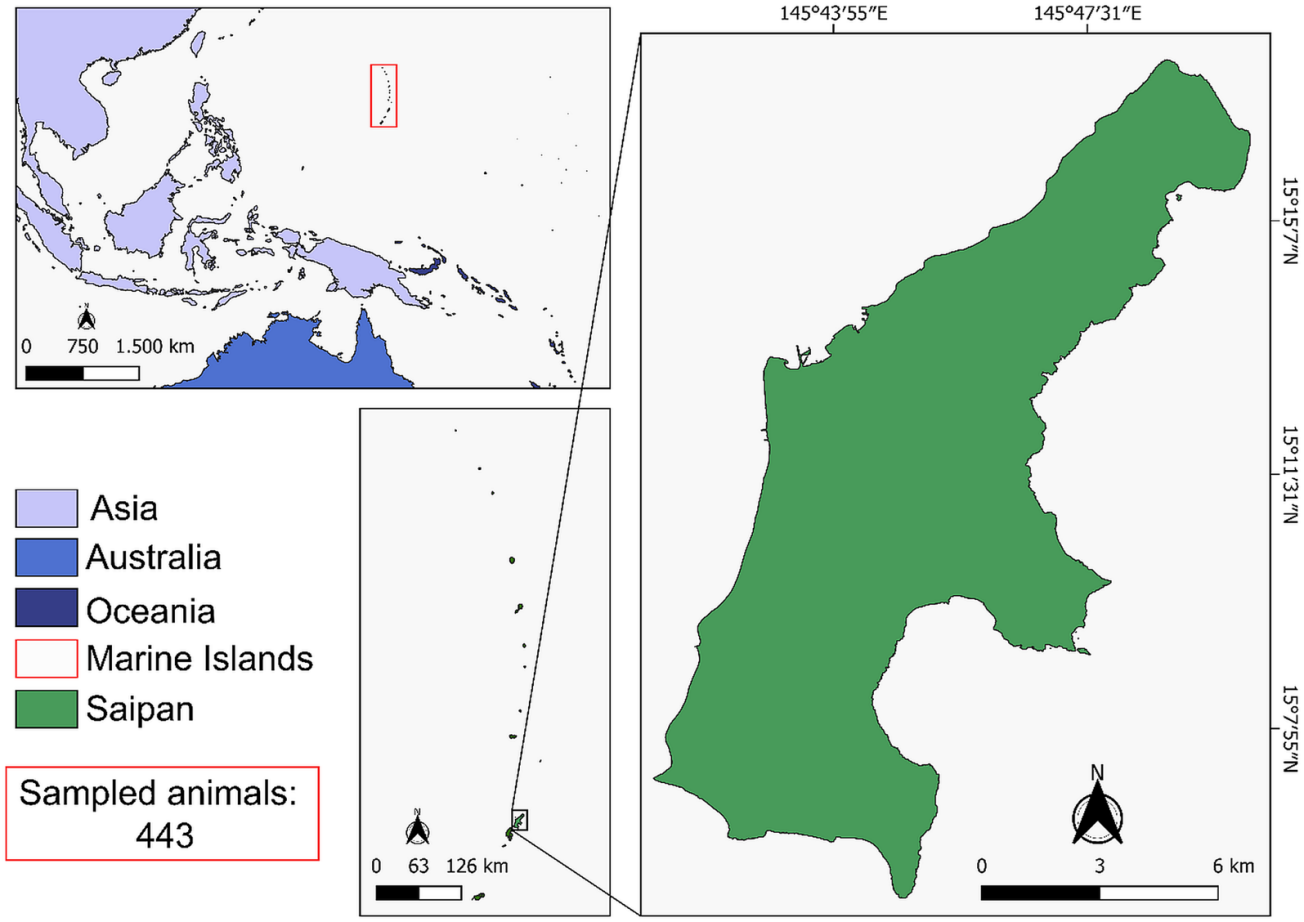
Aerial map of Saipan, Commonwealth of the Northern Mariana Islands

Saipan is the second largest island within the CNMI, with the second largest population within the 15-island chain [36]. The total land area is 119 km^2^ (15 ° 11′ 14″ N, 145 ° 44′ 49″ E) (Fig. 1) [36, 37]. Saipan has a tropical rainforest climate with little seasonal temperature variation and two seasons: wet and dry. The 2020 population of Saipan was estimated at 43,385 people, which is a decrease from the 2010 US estimate of 48,220 people [38].

**Fig. 2:**
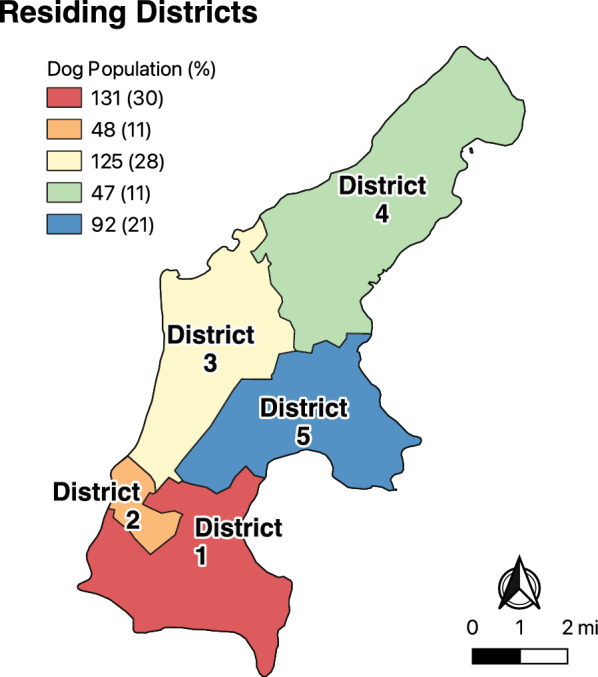
Aerial map of Saipan, Commonwealth of the Northern Mariana Islands, showing its election districts and subdivisions of the five election districts, retrieved from the 115th Congress of the US Census Bureau, 2023

The map (Fig. 1) was created using QGIS 3.28.15 software in which georeferenced data were inserted in continuous cartographic base maps (Shapefiles, version 2017), which are available from the database of the US Census Bureau. The residing district (Fig. 2) was created using QGIS 3.22.15 software using the continuous cartographic base maps (Shapefiles, version 2018) from the US Census Bureau database.

### Sampling and serological examination

Sampling was opportunistic and occurred during an island-wide spay-and-neuter event that took place from May 2023 to June 2023. The inclusion criteria consisted of dogs residing on Saipan that were enrolled in the spay-and-neuter event, regardless of age, sex, or breed. Dog owners and shelters were invited to participate during the spay-and-neuter event by the participating veterinarian. All dogs who participated in the spay-and-neuter event were included. Blood samples from 443 dogs were collected through venipuncture from either the cephalic or jugular vein and then stored in isothermal boxes (4 °C) until analysis. For each dog, owner-reported demographic data were recorded, including age, sex, residing district, and ownership status (Table 1). Dogs were grouped on the basis of the age reported by the owner or the estimated age by the attending veterinarian, with juveniles being ≤ 1 year old, adult dogs ranging from > 1–7 years old, and senior dogs being > 7 years old. In terms of ownership status, client-owned dogs were brought by an individual who self-identified as an owner. Dogs that were at a shelter at the time of sampling were considered either shelter dogs or owner-surrendered if admitted to the shelter within 2 days, on the basis of shelter records. The residing district followed the election districts as classified by the US Census Bureau, with districts one through five (Fig. 2) [39]. At the time of sampling for each dog, no clinical signs or prevention measures were recorded by the attending veterinarian.

**Table 1:** Demographics of the dog population sampled from Saipan, Commonwealth Northern Mariana Islands (*n* = 443)

Demographics	No. (%)
Age	
Juvenile	106 (23.9)
Adult	280 (63.2)
Senior	57 (12.8)
Sex	
Female	258 (58.2)
Male	185 (41.7)
Residing location	
District 1	131 (29.5)
District 2	48 (10.8)
District 3	125 (28.2)
District 4	47 (10.6)
District 5	92 (20.7)
Ownership status	
Client-owned	337 (76.0)
Owner-surrendered	50 (11.2)
Shelter	56 (12.6)

Using a commercial POC ELISA test (IDEXX SNAP® 4Dx® Plus test, IDEXX Laboratories, Westbrook, ME, USA), we assessed samples for the antigen of *D. immitis* and antibodies against *E. canis*, *E. chaffeensis*, *E. ewingii*, *A. phagocytophilum*, *A. platys*, and *B. burgdorferi* [40]. All samples were analyzed individually following manufacturer’s instructions. The sensitivity and specificity of this test for the *D. immitis* antigen is 98% [95% confidence interval (CI) 89.1–99.9%] and 100% (95% CI 99.2–100%), respectively [40]. The reported sensitivity and specificity for *Ehrlichia* spp. were 93.4% (95% CI 86.9–97.3%) and 96.8% (95% CI 94.6–98.3%), respectively [40]. Sensitivity and specificity for *Anaplasma* spp. was reported at 94% (95% CI 86.8–98.1%) and 98.4% (95% CI 96.6–99.3%), respectively [40]. Finally, the reported sensitivity and specificity for *B. burgdorferi* were 95% (95% CI 77.2–99.9%) and 99.4% (95% CI 98.2–99.9%), respectively [40].

### Data analysis

Pathogen prevalence, including single and co-detections, was determined using a generalized series interpretation that takes into account the calculation for the prevalence, adjusting for imperfect test sensitivity and specificity (Supplementary Data File 1) [41, 42]. The formulas make two major assumptions. The first assumption is that all detection tests for all detected pathogens are statistically independent. The second assumption is that a test is independent of the true status of the other diseases, with no interference in the sensitivity and specificity of the test. The calculated sensitivities and specificities used as a basis for each pathogen in the formula were based on those reported for the IDEXX SNAP^®^ 4Dx^®^ test by the manufacturer [40].

Statistical analysis was performed using STATA version 18.0 BE-Basic Edition (Stata, College Station, Texas, USA). Chi-squared tests were used to perform a univariate analysis assessing the association between pathogen prevalence and the evaluated potential risk factors. If there was an association with *P* ≤ 0.2, the factor was included in a multivariable logistic regression analysis using a backward stepwise selection process [43]. A cutoff of *P* ≤ 0.2 was used to determine statistical significance. This *P*-value was selected because of this study’s exploration and determination of potential trends of pathogen detection for future investigations. We performed the Hosmer–Lemeshow and Pearson *χ*^2^ tests to determine how well each model fit the observed data [41]. We then generated receiver operating characteristic (ROC) curves for each multivariable logistic regression model during a single evaluation to understand each model’s performance. Then, a ten-fold cross validation ROC curve was generated to provide a more comprehensive view of each model [43].

## Results

We sampled 443 dogs residing on the island of Saipan. The majority of tested dogs were adults (>1 year old to 7 years old), making up 63.2% of the sampled population. Female dogs made up 58.2% of the population, while male dogs made up 41.7%. Dogs from all electoral districts were represented in these samples. District one made up 29.5% of the sampled population. Most sampled dogs (76.0%) were client-owned (Table 1).

Overall, 66.16% (*n* = 300/443; exact 95% CI 60.17–70.55) of dogs were positive for at least one pathogen. These included *Ehrlichia* spp. with 58.0% (*n* = 246/443), followed by 43.1% (184/443) for *Anaplasma* spp. and *D. immitis* having 14.7% (*n* = 64/443). A single pathogen was detected in 137 dogs. Antibodies against *Ehrlichia* spp. were detected in 21.67% (*n* = 89/443; exact 95% CI 17.83–27.07) of the samples. This was followed by antibodies against *Anaplasma* spp. in 7.68% (*n* = 32/443; exact 95% CI 5.22–10.94) of dogs. Finally, the *D. immitis* antigen was detected in 3.87% (*n* = 16/443; exact 95% CI 2.08–6.21) of dogs. We did not detect *B. burgdorferi* antibodies in the dogs sampled in this study (Table 2).

**Table 2:** Seropositivity for each canine vector-borne pathogen in the sampled population

Pathogen	No. positive	Prevalence (%)	Wald 95% CI	Exact 95% CI
*D. immitis*	16	3.87	1.99–5.75	2.08–6.21
*Ehrlichia* spp.	89	21.67	17.22–26.12	17.83–27.07
*Anaplasma* spp.	32	7.68	4.95–10.41	5.22–10.94
*B. burgdorferi*	–	–	–	–
Co-detections of two pathogens				
*Ehrlichia* spp. + *Anaplasma* spp.	115	29.34	23.79–34.89	24.68–36.17
*Ehrlichia* spp. + *D. immitis*	11	2.76	1.13–4.39	1.18–4.90
*Anaplasma* spp. + *D. immitis*	6	1.52	0.31–2.73	0.26–3.24
Co-detection of three pathogens				
*Ehrlichia* spp. + *Anaplasma* spp. + *D. immitis*	31	8.14	5.23–11.05	5.53–11.68
Total pathogens detected				
*D. immitis*	64	14.75	11.36–18.14	11.70–18.80
*Ehrlichia* spp.	246	58.01	51.99–64.03	52.48–64.78
*Anaplasma* spp.	184	43.14	37.61–48.67	38.11–49.46
Total	300	66.16	61.16–71.16	60.17–70.55

The most common co-detections were *Ehrlichia* spp. + *Anaplasma* spp. (29.34%; *n* = 115/443; exact 95% CI 24.68–36.17) followed by *Ehrlichia* spp. + *D. immitis* (2.76%; *n* = 11/443; exact 95% CI 1.18–4.90) and *Anaplasma* spp. + *D. immitis* (1.52%; *n* = 6/443; exact 95% CI 0.26–3.24). Co-detection of three pathogens, *Ehrlichia* spp. + *Anaplasma* spp. + *D. immitis*, was also observed (8.14%; *n* = 31/443; exact 95% CI 5.53–11.68).

The results of the univariate analysis of risk factors are presented in Table 3. *D. immitis* was significantly associated with age (*P* < 0.001), residing districts (*P* = 0.001), and ownership status (*P* < 0.001). *Ehrlichia* spp. was significantly associated with age (*P* < 0.001), residing districts (*P* = 0.177), and ownership status (*P* = 0.014). Finally, *Anaplasma* spp. was significantly associated with ownership status (*P* < 0.001).

**Table 3:** Risk factors associated with seroprevalence of canine vector-borne pathogens among dogs in Saipan

Variable	Total no.	*D. immitis*			*Ehrlichia* spp.			*Anaplasma* spp.		
		No. (%)	95% CI	Chi-squared *df* *P*-value	No. (%)	95% CI	Chi-squared *df* *P*-value	No. (%)	95% CI	Chi-squared *df* *P*-value
Age										
Juvenile (≤ 1 year old)	106	4 (3.7)	0.01–0.09	$\chi$ ^2^ = 20.534 *df *= 2 ***P* = < 0.001**	35 (33.0)	24.1–42.8	$\chi$^2^ = 37.103 *df *= 2 ***P* = < 0.001**	37 (34.9)	25.9–44.7	$\chi$^2^ = 2.918 *df* = 2 *P* = 0.232
Adult (> 1–7 years old)	280	55 (19.6)	15.1–24.7		166 (59.2)	53.2–65.0		120 (42.8)	36.9–48.8	
Senior (> 7 years old)	57	8 (8.7)	2.9–19.2		45 (78.9)	66.1–88.6		27 (47.3)	33.9–61.0	
Sex										
Male	185	37 (14.3)	10.3–19.2	$\chi$^2^ = 0.005 *df* = 1 *P* = 0.940	102 (55.1)	47.6–62.4	$\chi$^2^ = 0.020 *df* = 1 *P* = 0.887	102 (55.1)	47.6–62.4	$\chi$^2^ = 2.945 *df* = 1 *P* = 0.229
Female	258	27 (14.5)	9.8–20.5		144 (55.8)	49.5–61.9		105 (40.6)	31.2–42.7	
Residing district										
District 1	131	16 (12.2)	7.1–19.0	$\chi$^2^ = 19.011 *df* = 4 ***P* = 0.001**	70 (53.4)	44.5–62.1	$\chi$^2^ = 6.312 *df* = 4 ***P* = 0.177**	53 (40.4)	31.9–49.3	$\chi$^2^ = 2.722 *df* = 2 *P* = 0.602
District 2	48	1 (0.02)	0.05–11.0		25 (52.0)	37.1–66.7		21 (43.7)	29.4–58.8	
District 3	125	15 (12.0)	6.8–19.0		62 (49.6)	40.5–58.6		47 (37.6)	29.0–46.7	
District 4	47	14 (29.7)	17.3–44.8		30 (63.8)	48.5–77.3		24 (51.0)	36.0–65.9	
District 5	92	18 (19.5)	12.0–29.1		59 (64.1)	53.4–73.8		39 (42.3)	32.1–53.1	
Ownership status										
Client-owned	337	28 (8.3)	5.5–11.7	$\chi$^2^ = 37.314 *df* = 2 ***P* = < 0.001**	175 (51.9)	46.4–57.3	$\chi$^2^ = 8.605 *df* = 2 ***P* = 0.014**	123 (36.4)	31.3–41.8	$\chi$^2^ = 15.738 *df* = 2 ***P* = < 0.001**
Owner-surrendered	50	18 (36.0)	22.9–50.8		31 (62.0)	47.1–75.3		26 (52.0)	37.4–66.3	
Shelter	56	18 (32.1)	20.2–45.9		40 (71.4)	57.7–82.7		35 (62.5)	48.5–75.0	

Significant relationships (*P* < 0.2) denoted by bold font

(*df* degrees of freedom)

Multivariable logistic regression results are found in Tables 4, 5, and 6 for *D. immitis*, *Ehrlichia* spp., and *Anaplasma* spp., respectively. Juvenile dogs had 88% lower odds of testing positive for *D. immitis* (*P* < 0.001, 95% CI 0.04–0.37) than adult dogs, owner-surrendered dogs had 7.34 times higher odds (*P* < 0.001, 95% CI 3.29–16.34), and shelter dogs had 6.35 times higher odds (*P* < 0.001, 95% CI 2.94–13.71) of testing positive for *D. immitis* than owned dogs. Dogs from district five had 2.13 times higher odds (*P* = 0.072, 95% CI 0.93–4.85), dogs from district four had 3.76 times higher odds (*P* = 0.005, 95% CI 1.49–9.45), and dogs from district two had 85% lower odds (*P* = 0.078, 95% CI 0.01–1.23) of testing positive for *D. immitis* than dogs from district one. We found that the Hosmer–Lemeshow (*P* = 0.01) and Pearson *χ*^2^ (*P* = 0.03) tests were statistically significant, providing evidence that the model did not fit the observed data well. However, we did find that the area under the curve (AUC) for a single evaluation was 0.80, which indicates considerable accuracy (Supplementary Fig. 2) [44]. When utilizing the tenfold cross validation, we found the AUC was 0.77, which shows that the accuracy was fair and not badly overfit for the final model (Supplementary Fig. 3) [44].

**Table 4:** Association of *D. immitis* with potential risk factors

*D. immitis*	OR	SE	*Z*	*P*	95% CI
Age					
Adult	*	*	*	*	*
Juvenile	0.128	0.070	−3.71	** < 0.001**	0.043–0.379
Senior	0.670	0.358	−0.75	0.455	0.235–1.91
Ownership status					
Client-owned	*	*	*	*	*
Owner-surrendered	7.341	2.998	4.88	** < 0.001**	3.297–16.346
Shelter	6.353	2.494	4.71	** < 0.001**	2.942–13.715
Residing district					
One	*	*	*	*	*
Five	2.131	0.896	1.80	**0.072**	0.935–4.858
Four	3.761	1.769	2.82	**0.005**	1.495–9.459
Three	0.913	0.379	−0.22	0.827	0.404–2.060
Two	0.151	0.162	−1.76	**0.078**	0.018–1.233
Intercept	0.094	0.032	−6.85	< 0.001	0.047–0.185

Significant relationships (*p* < 0.2) denoted by bold font

*OR* odds ratio, *SE* standard error; *Z* *Z* statistic, *P* *P*-value, *CI* confidence interval, * reference category

**Table 5:** Association of *Ehrlichia* spp. with potential risk factors

*Ehrlichia* spp.	OR	SE	*Z*	*P*	95% CI
Age					
Adult	*	*	*	*	*
Juvenile	0.311	0.083	−4.34	** < 0.001**	0.0183–0.527
Senior	3.158	1.176	3.09	**0.002**	1.521–6.556
Ownership status					
Client-owned	*	*	*	*	*
Owner-surrendered	1.707	0.613	1.49	**0.137**	0.844–3.451
Shelter	1.982	0.704	1.93	**0.054**	0.987–3.980
Coinfections					
*Anaplasma* spp.	5.946	1.406	7.54	** < 0.001**	3.740–9.454
Intercept	0.640	0.103	−2.74	0.006	0.466–0.879

Significant relationships (*P* < 0.2) denoted by bold font

*OR* odds ratio, *SE* standard error, *Z* *Z* statistic, *P* *P*-value, *CI* confidence interval, * reference category

**Table 6:** Association of *Anaplasma* spp. with potential risk factors

*Anaplasma* spp.	OR	SE	*Z*	*P*	95% CI
Ownership status					
Client-owned	*	*	*	*	*
Owner-surrendered	1.537	0.527	1.25	0.210	0.784–3.012
Shelter	2.171	0.719	2.34	**0.019**	1.134–4.156
Coinfections					
*Ehrlichia* spp.	5.776	1.300	7.79	** < 0.001**	3.715–8.981
*D. immitis*	1.616	0.510	1.53	**0.129**	0.870–3.002
Intercept	0.198	0.038	−8.33	< 0.001	0.135–0.289

Significant relationships (*P* < 0.2) denoted by bold font

*OR* odds ratio, *SE* standard error, *Z* *Z* statistic, *P* *P*-value, *CI* confidence interval, * reference category

Juvenile dogs had 69% lower odds (*P* < 0.001, 95% CI 0.01–0.52), while senior dogs had 3.15 times higher odds (*P* = 0.002, 95% CI 1.52–6.55) of testing positive for *Ehrlichia* spp. than adult dogs. Dogs who were owner-surrendered had 1.70 times higher odds (*P* = 0.137, 95% CI 0.84–3.45), while shelter dogs had 1.98 times higher odds (*P* = 0.054, 95% CI 0.98–3.98) of testing positive for *Ehrlichia* spp. compared with owned dogs. Dogs who were positive for *Anaplasma* spp. had 5.94 times higher odds (*P* < 0.001, 95% CI 3.74–9.45) of testing positive for *Ehrlichia* spp. as a co-detection. For the goodness-of-fit, we found that the Hosmer–Lemeshow (*P* = 0.78) and Pearson *χ*^2^ (*P* = 0.84) tests were not significant, showing that there was no evidence of a lack of fit. We found that the single evaluation AUC for the ROC curve was 0.77, which has a fair accuracy (Supplementary Fig. 4) [44]. Moreover, the tenfold cross validation AUC was 0.76, showing fair accuracy as well (Supplementary Fig. 5) [44].

Shelter dogs had 2.17 times higher odds (*P* = 0.019, 95% CI 1.13–4.15) of testing positive for *Anaplasma* spp. than owned dogs. Dogs that were positive for *Ehrlichia* spp. had 5.77 times higher odds (*P* < 0.001, 95% CI 3.71–8.98) of testing positive for *Anaplasma* spp., adjusting for ownership status, and dogs that tested positive for *D. immitis* had 1.61 times higher odds (*P* = 0.129, 95% CI 0.87–3.00) of testing positive for *Anaplasma* spp. For this model, the Hosmer–Lemeshow (*P* = 0.11) and Pearson *χ*^2^ (*P* = 0.25) tests were not significant, meaning no evidence of a lack of fit. We found that the single evaluation ROC curve AUC was 0.74, which shows fair accuracy (Supplementary Fig. 6) [44]. In addition, the tenfold cross validation AUC was 0.72 showing fair accuracy as well (Supplementary Fig. 7) [44].

## Discussion

This study reports on the exposure to VBPs in dogs that inhabit an insular area in which a limited number of studies are available in scientific literature. The overall seropositivity of CVBP was 66.1% (*n* = 300/443) in this study. This indicates that dogs living in this area are highly exposed to CVBPs. The most commonly detected was *Ehrlichia* spp. (58.0%; *n* = 246/443), followed by *Anaplasma* spp. (43.1%; *n* = 184/443) and *D. immitis* (14.7%; *n* = 64/443). We found that there was a high seroprevalence in co-detections between *Ehrlichia* spp. + *Anaplasma* spp. (29.34%). In remote areas, preventive measures against ecto- and endoparasites are not usually adopted, likely owing to the reduced perception of the risk for dogs, as well as limited access to veterinary healthcare [45].

The high positivity observed for *Ehrlichia* spp. (21.67%) and *Anaplasma* spp. (7.68%) indicates that these animals are exposed to tick infestations, most likely *R. sanguineus* s.l. [46]. These CVBPs have been reported worldwide, with a large overlap in distribution owing to their shared brown dog tick vector [2]. Despite the absence of studies on dogs, the previous detection of *Anaplasma* spp. and *E. canis* DNA in ticks from this area indicated the potential risk to which animals were exposed [47]. Although *B. burgdorferi* was not detected in this study, it does not exclude its presence in the study area. The absence could be partially related to the vector competence of ticks infecting these animals or owing to the number of animals sampled [48].

We also detected the*D. immitis* antigen in these dogs, which demonstrates an active heartworm infection at the time of sampling [40, 49]. The presence of *D. immitis* on Saipan is not surprising, as this parasite has been reported in various regions of the Pacific Southwest, including historically and geopolitically related areas such as neighboring Guam, the Philippines, and the continental USA [49–52]. Molecular phylogenetic investigations on *D. immitis* isolates circulating on the island could shed light on origin and test hypotheses around parasite introduction. In addition, suitable mosquito vectors are known to occur on Saipan, including *Aedes*, *Anopheles*, and *Culex* species, yet they remain unknown with regard to their epidemiological role in *D. immitis* transmission [53]. Despite heartworm antigen being found in 14.8% of the dogs, some infections could have been missed (i.e., false negative results) [54]. One way to overcome this potential issue is through the incorporation of immune complex disassociation (ICD) via heat treatment to free the *D. immitis* antigen that could be bound to host antibodies [54–57]. This has been shown to improve detection of active infections of heartworm in dogs living in endemic areas with an unknown chemoprophylaxis status and may also detect infections earlier than 6–7 months [11, 55, 57, 58]. Most veterinary advisory boards recommend a combination of both antigen-detection and microscopy-based methods to diagnose heartworm infection in dogs, and the addition of molecular diagnostics can aid in more specific detection, which should be included in future studies [11, 19, 22, 56, 59–61]. Owing to logistical constraints in this study, only heartworm antigen testing could be performed at the time of sampling. The incorporation of other diagnostic tests can also allow for the detection of single or multiple detections with other filarial nematodes [62].

A high prevalence of CVBPs has been found on other Pacific islands in proximity to those sharing similar climates [47]. For instance, in a previous study with 136 dogs, a similar pattern in tick-borne pathogens was observed on Guam, including the detection of antibodies against *Ehrlichia* spp. (14.7%) and *Anaplasma* spp. (31.6%) and molecular detection of *A. platys* (19.1%), *A. phagocytophilum* (5.9%), and *E. canis* (12.5%) [47]. There are few data available on CVBPs across most Pacific Islands, but in the few published reports, similar patterns to the present study have been documented [47, 63]. This could be because of the same transmitting vector and transmission via bloodmeal [63]. There has also been detection in other areas outside of the Pacific Islands, which show similar co-detections found in this study, specifically in Asia, Africa, and island groups located on the Atlantic Ocean [64–67].

In this study, we detected a high prevalence of antibody detection against *Anaplasma* spp., and *Ehrlichia* spp. This antibody detection focuses on a highly specific set of antibodies that can only be found if a dog has been exposed to a pathogen at some point prior to sampling [40]. Considering the high prevalence, it can be beneficial to analyze the molecular characterization of those infected with both species because of the zoonotic potential found in certain species of *Ehrlichia* and *Anaplasma* [26, 33–35].

On the basis of the multivariable logistic regression, owner-surrendered dogs had a higher likelihood of testing positive for both *D. immitis* and *Ehrlichia* spp. compared with client-owned dogs. This could be owing to access and affordability of prevention drugs and veterinary care. It was also observed that shelter dogs had a higher likelihood of testing positive for all three pathogens, which has also been documented across similar studies completed in the continental USA [68–70]. This is also noteworthy owing to limited availability of prevention measures and annual screening in shelters.

Animal movement from and to the USA has been seen in client-owned dogs, especially dogs that are highly trained for specified tasks, such as military, Transportation Security Administration (TSA), or even service dogs. This movement poses an increased risk for transmission by dogs that can serve as sentinels for many VBPs [71]. Working dogs have the potential to travel to nonendemic regions and introduce new pathogens. Nevertheless, all detected CVBPs found on Saipan are known to occur in the continental USA and other countries [51, 64, 72–76]. However, there is a risk of novel pathogens that may not be currently present on the island to be introduced through these working dogs, warranting broad pathogen surveillance.

All samples used in this study were opportunistically collected through an island-wide spay-and-neuter event. Therefore, there are some limitations related to the absence of data such as clinical signs, prevention use, unknown living situation (living outside versus indoors), and travel history. In three pathogens found in this study, asymptomatic infections may have been relatively common at the time of diagnosis; therefore, a more thorough evaluation for clinical signs and a detailed history of the use of preventive medications would have been informative. Although the living conditions and lifestyles of the dogs were not reported in the present study, it is known that most owned dogs residing on Saipan are housed indoors compared with fenced, chained, or free-to-roam dogs[77]. These factors can uncover the risks associated with CVBPs found in this canine population. It is also worth noting that there was a limitation within the final model, *D. immitis*. Although both goodness-of-fit tests were significant, they provided evidence that the final model did not fit the data well. However, we did find that the area under the curve for both a single evaluation (AUC = 0.80) and tenfold evaluation (AUC = 0.77) showed that the accuracy interpretation was considerable and fair (Supplementary Figs. 2 and 3) [44]. Despite that, the data presented herein are the first available from Saipan and will serve as a baseline for further studies.

## Conclusions

This study reported a high frequency of CVBDs in a dog population from Saipan, CNMI, an area with an unknown epidemiological status. The seroprevalence for these pathogens, including co-detections, shows that active surveillance can provide information on pathogen distribution and risk factors association in an understudied, geographically remote canine population. The results of this study emphasize the need to raise awareness of CVBPs, increase access to prevention measures, and implement control measures.

## Supplementary Information

Supplementary Material 1.

## Data Availability

Data are provided within the manuscript or Supplementary Information files.
