# Supplementary material for: High seroprevalence of selected vector-borne pathogens in dogs from Saipan, Northern Mariana Islands
Source: Parasit Vectors. 2025 Feb 24;18:75. doi: 10.1186/s13071-025-06705-2 (PMC11853585; doi:10.1186/s13071-025-06705-2)
Supplement: Supplementary file 1 — Supplementary Material 1. [file 13071_2025_6705_MOESM1_ESM.docx]

**Supplementary Data 1:**

A generalization of the series interpretation for test results.

Major assumption:

1. all three tests are statistically independent.
2. A test is independent of the true status for the other diseases. (not interfering with the sensitivity/specificity of the test)

Se: Sensitivity; Sp: Specificity; H: heartworm Ag; E: *Ehrlichia* Ab; A: *Anaplasma* Ab

**Three-way coinfections (series interpretation):**

Sensitivity: Se_H_ * Se_E_ * Se_A_

Specificity: Sp_H_ + Sp_E_ + Sp_A_ – (Sp_H_ * Sp_E_) – (Sp_H_ * Sp_A_) – (Sp_E_ * Sp_A_) + (Sp_H_ * Sp_E_ * Sp_A_)

**Two-way coinfections (Heartworm and *Ehrlichia*)**

Sensitivity: Se_H_ * Se_E_ * Sp_A_

Specificity: Sp_H_ + Sp_E_ + Se_A_ – (Sp_H_ * Sp_E_) – (Sp_H_ * Se_A_) – (Sp_E_ * Se_A_) + (Sp_H_ * Sp_E_ * Se_A_)

**Two-way coinfections (Heartworm and *Anaplasma*)**

Sensitivity: Se_H_ * Se_A_ * Sp_E_

Specificity: Sp_H_ + Sp_A_ + Se_E_ – (Sp_H_ * Sp_A_) – (Sp_H_ * Se_E_) – (Sp_A_ * Se_E_) + (Sp_H_ * Sp_A_ * Se_E_)

**Two-way coinfections (*Ehrlichia* and *Anaplasma*)**

Sensitivity: Se_E_ * Se_A_ * Sp_H_

Specificity: Sp_E_ + Sp_A_ + Se_H_ – (Sp_E_ * Sp_A_) – (Sp_E_ * Se_H_) – (Sp_A_ * Se_H_) + (Sp_E_ * Sp_A_ * Se_H_)

**Single infection (Heartworm)**

Sensitivity: Se_H_ * Sp_E_ * Sp_A_

Specificity: Sp_H_ + Se_E_ + Se_A_ – (Sp_H_ * Se_E_) – (Sp_H_ * Se_A_) – (Se_E_ * Se_A_) + (Sp_H_ * Se_E_ * Se_A_)

**Single infection (*Ehrlichia*)**

Sensitivity: Se_E_ * Sp_H_ * Sp_A_

Specificity: Sp_E_ + Se_H_ + Se_A_ – (Sp_E_ * Se_H_) – (Sp_E_ * Se_A_) – (Se_H_ * Se_E_) + (Sp_E_ * Se_H_ * Se_A_)

**Single infection (*Anaplasma*)**

Sensitivity: Se_A_ * Sp_E_ * Sp_H_

Specificity: Sp_A_ + Se_E_ + Se_H_ – (Sp_A_ * Se_E_) – (Sp_A_ * Se_H_) – (Se_E_ * Se_H_) + (Sp_A_ * Se_E_ * Se_H_)

**Overall infection (parallel interpretation)**

Sensitivity: Se_A_ + Se_E_ + Se_H_ – (Se_A_ * Se_E_) – (Se_A_ * Se_H_) – (Se_E_ * Se_H_) + (Se_A_ * Se_E_ * Se_H_)

Specificity: Sp_A_ * Sp_E_ * Sp_H_

**Supplementary Figure 2:** Receiver operating characteristic curve to evaluate the performance for final model of *Dirofilaria immitis* at possible cutpoints.


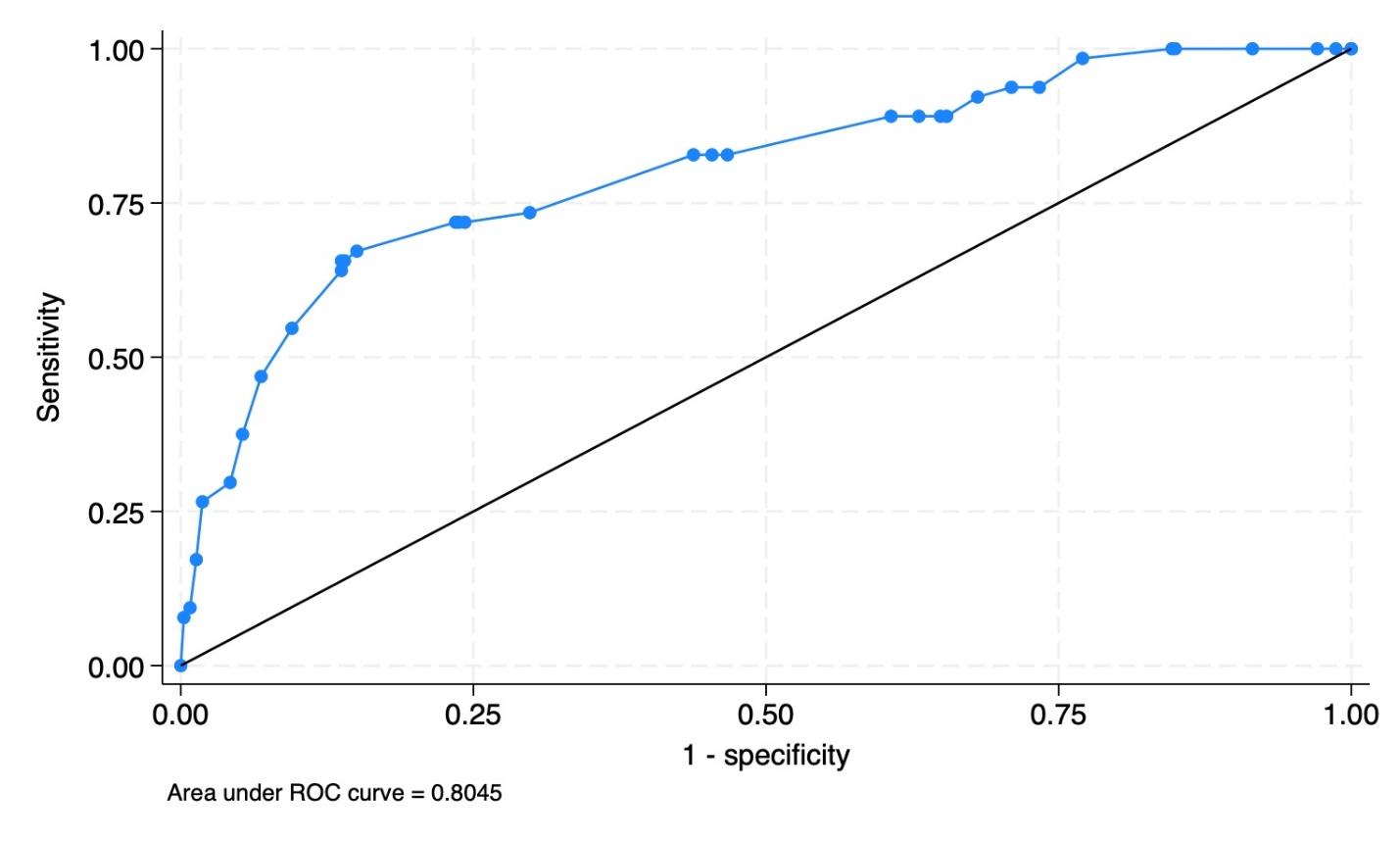


**Supplementary Figure 3:** Receiver operating characteristic curve using a ten-fold cross validation to evaluate the accuracy of the final model of *Dirofilaria immitis.*


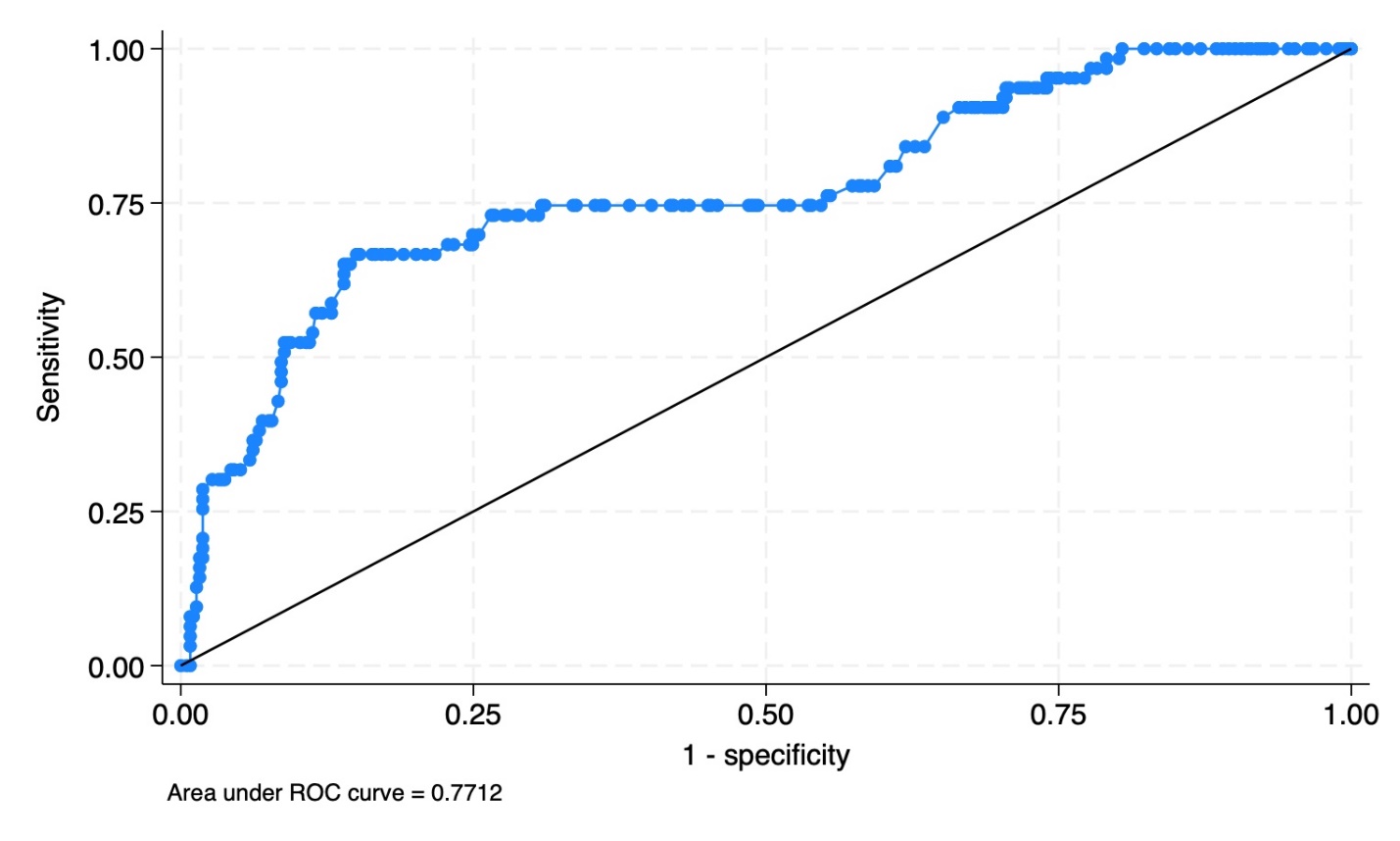


**Supplementary Figure 4:** Receiver operating characteristic curve to evaluate the performance for final model of *Ehrlichia* spp. at possible cutpoints.

**
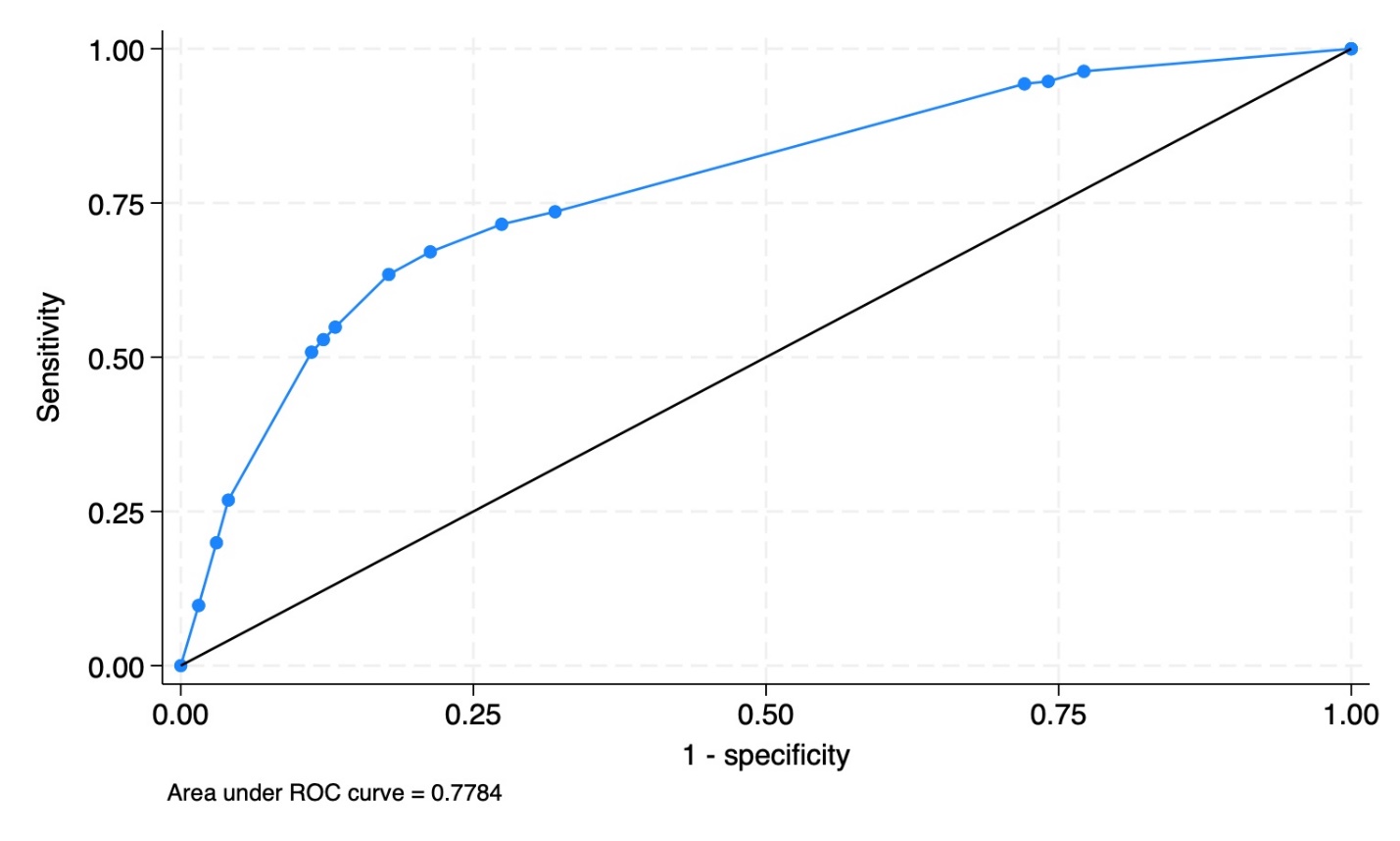
**

**Supplementary Figure 5:** Receiver operating characteristic curve using a ten-fold cross validation to evaluate the accuracy of the final model of *Ehrlichia* species.

**
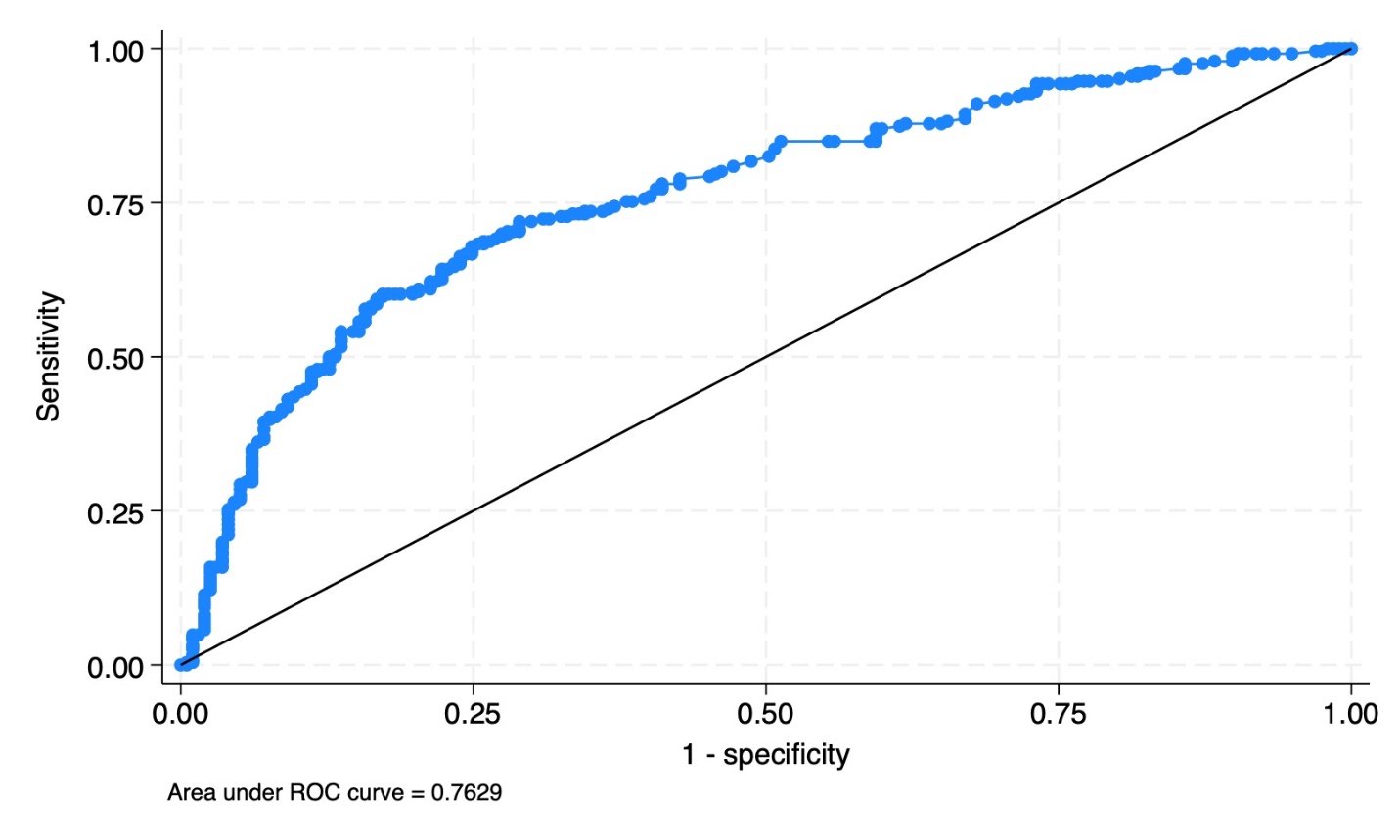
**

**Supplementary Figure 6:** Receiver operating characteristic curve to evaluate the performance for final model of *Anaplasma* spp. at possible cutpoints**.**

**
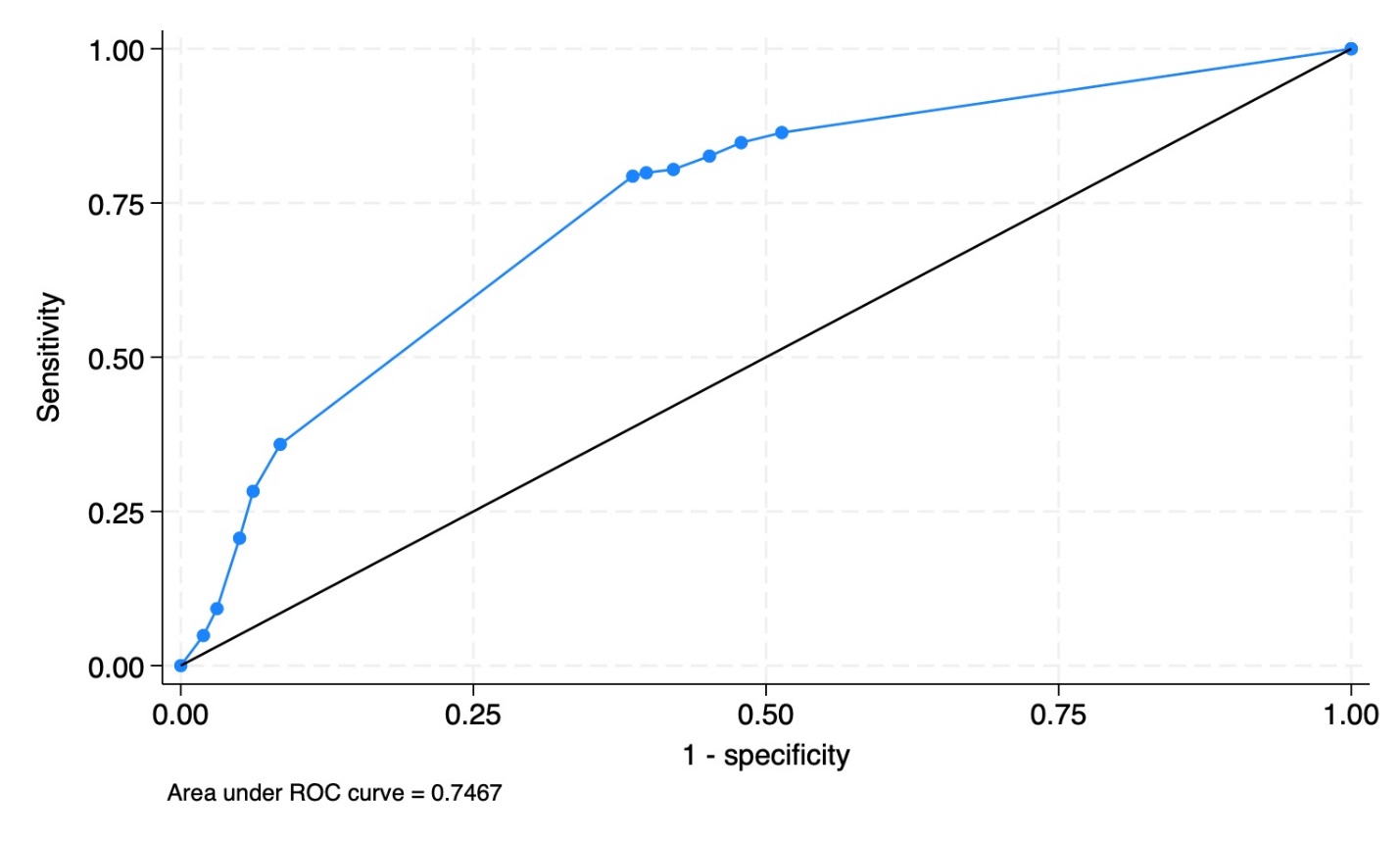
**

**Supplementary Figure 7:** Receiver operating characteristic curve using a ten-fold cross validation to evaluate the accuracy of the final model of *Anaplasma* species.

**
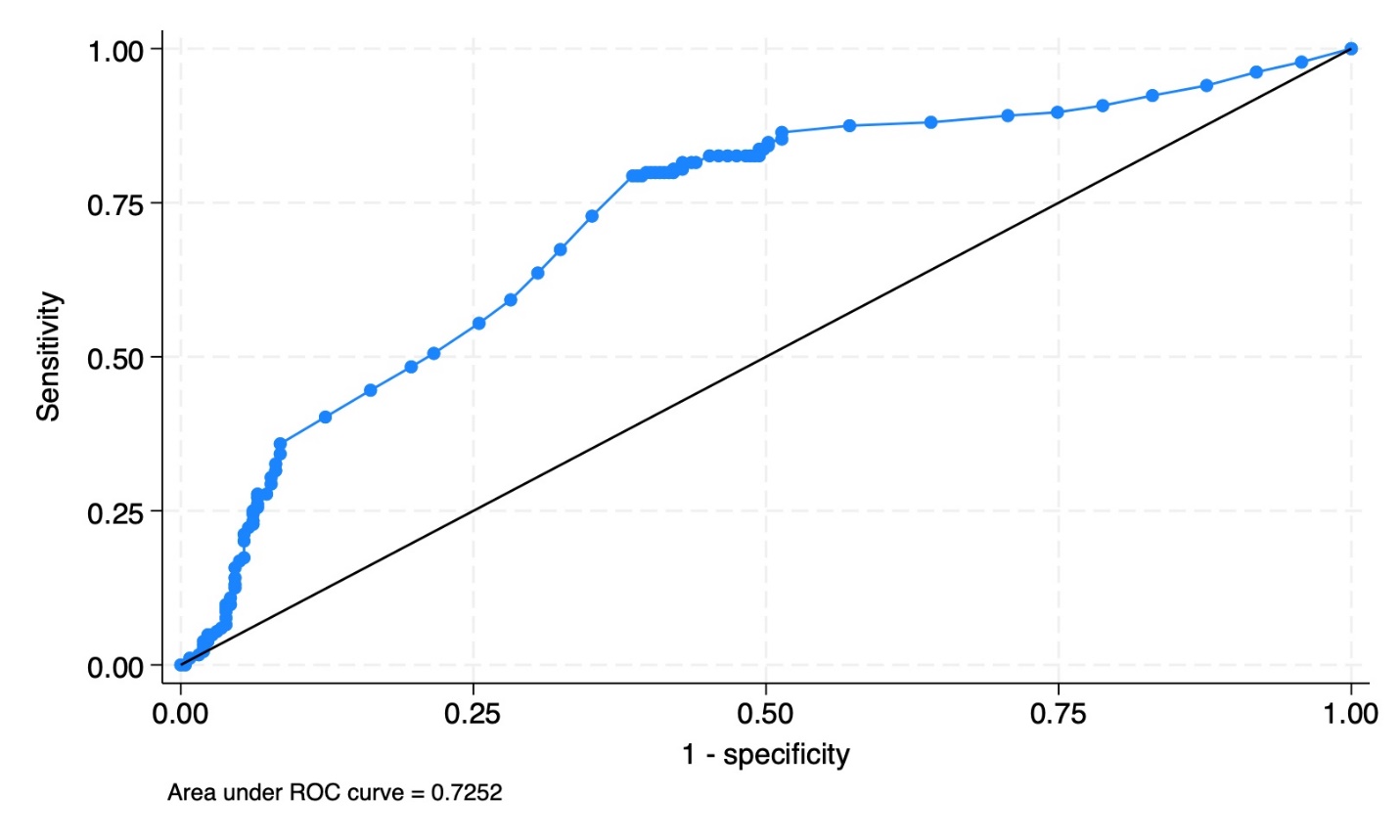
**
